# On the potential of group-specific component (GC) inhibition for treating metabolic disease

**DOI:** 10.20517/mtod.2025.195

**Published:** 2026-03-04

**Authors:** Richard Gill, Taiyi Kuo

**Affiliations:** Department of Neurobiology, Physiology, and Behavior, University of California Davis, Davis, CA 95616, USA.

**Keywords:** Metabolism, obesity, diabetes, group-specific component, vitamin D binding protein, insulin secretion, insulin sensitivity, therapeutics

## Abstract

Recent advances in obesity and type 2 diabetes treatment using glucagon-like peptide-1 receptor agonist (GLP1RA)-based therapeutics have enabled major improvements in the management of these metabolic disorders. However, many patients cannot tolerate side effects associated with appetite suppression, and around 10% of those adhering to treatment do not lose weight. Thus, there is a need for alternative and/or additional treatment. Here, we examine the therapeutic potential of targeting a gene called *Group-specific component* (*GC*) to treat metabolic diseases. First, we review GC’s established roles in vitamin D transport, metabolism, and inflammation, including its structure-activity relationships as well as the phenotypes associated with genetic variation in *GC*. Next, we summarize studies of *GC*-null humans and mice, which were both generally healthy and viable, demonstrating the safety of inhibiting GC. Finally, we discuss novel evidence that among mice fed a high fat diet, *Gc* ablation confers protection against the development of obesity and type 2 diabetes. Notably, these benefits came without reductions in food intake or lean mass, the primary drivers of GLP1RA-associated adverse effects. GC therefore represents a promising novel therapeutic target for metabolic diseases.

## Graphical Abstract

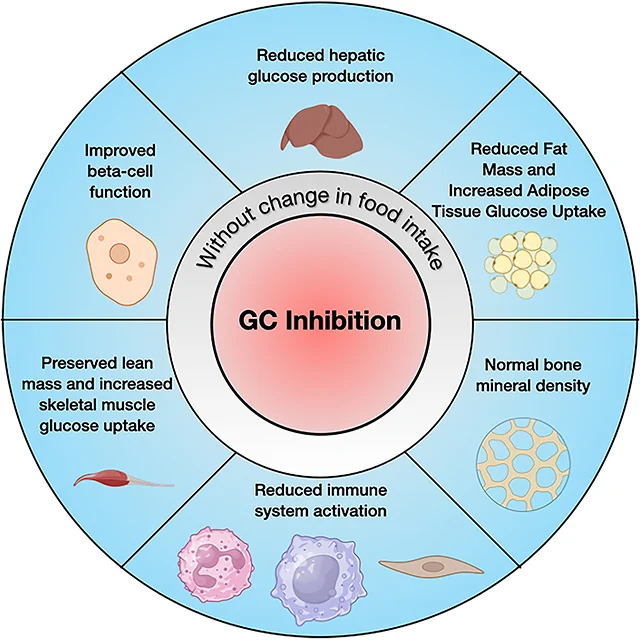

## INTRODUCTION

### Background

Obesity and type 2 diabetes are driven by inadequate exercise, poor dietary habits, and genetic predisposition, and are major causes of morbidity and mortality worldwide. Defined as having excess adipose tissue resulting in a disproportionate ratio of body weight relative to height [body mass index (BMI) ≥ 30 kg/m^2^], obesity affects over 40% of US adults^[1]^, and is a major risk factor for type 2 diabetes, cardiovascular diseases, certain forms of cancer, and all-cause mortality^[2]^. Both obesity and sedentary lifestyle contribute to insulin resistance, particularly in the liver, skeletal muscle, and adipose tissue. As a result of insulin resistance, there is an increased demand for insulin, and insulin-secreting pancreatic beta-cells’ failure to expand and compensate results in progression to hyperglycemia (high blood sugar concentration), the hallmark of type 2 diabetes^[3]^.

Prior to overt hyperglycemia and a formal type 2 diabetes diagnosis is prediabetes or intermediate hyperglycemia^[4]^, defined as a high-risk state characterized by one of the following criteria: (1) elevated fasting glucose levels (100–125 mg/dL) due to the liver’s resistance to insulin’s suppression of hepatic glucose production; (2) impaired glucose tolerance (based on an oral glucose tolerance test) due to insulin resistance in skeletal muscle, resulting in reduced postprandial glucose uptake and consequent elevated blood glucose (140 to 199 mg/dL); and (3) increased levels of glycated hemoglobin A1c (HbA1c; 5.7%–6.4%) representing the proportion of hemoglobin beta-chains with non-enzymatically added glucose.

Nutrient-stimulated hormones such as glucagon-like peptide-1 (GLP-1) and gastric inhibitory peptide (GIP) promote insulin secretion in metabolically healthy individuals, and their receptors have been targeted by GLP-1 receptor agonist (GLP1RA)-based therapeutics that have revolutionized the management and treatment of type 2 diabetes and obesity. Remarkably, when combined with diet and exercise, these treatments can result in good glycemic control and up to 20% body weight loss^[5]^; for the first time in over 10 years, the prevalence of obesity in the US decreased in 2023^[6]^. However, these medications suppress appetite and have adverse effects such as nausea, vomiting, diarrhea, constipation, stomach pain, and bloating, as well as muscle and bone loss^[5,7,8]^. Among obese patients who can tolerate these drugs, around 10% do not lose weight, or only lose weight initially before their weight “plateaus”^[5,8]^. Next-generation therapeutics are needed to address present adverse effects and non-response associated with GLP1RA treatment for obesity and type 2 diabetes. In this review we discuss the therapeutic potential of inhibiting Group-specific component (GC), also known as vitamin D binding protein, which by virtue of its distinct mechanism of action on metabolic health demonstrates potential to be a promising target for therapeutic intervention that could confer the benefits of GLP1RAs without the known side effects.

### GC (vitamin D binding protein)

GC is a multifunctional circulating protein and a member of the albumin superfamily of binding proteins which includes albumin, alpha-albumin, and alpha-fetoprotein^[9]^. The mature form of human GC protein is made up of 458 amino acids (AAs), and is primarily expressed in the liver, where it is synthesized into a ~58 kDa single-chain glycoprotein^[10]^. GC was named “group-specific” due to codominant non-synonymous genetic variants which governed the degree to which carriers’ serum GC migrated toward the anode (+) in gel electrophoresis studies: GC1F (fast) and GC1S (slow) [due to rs7041 affecting AA 416 in the mature protein], and GC2 [rs4588 affecting AA 420 in the mature protein]^[11–13]^ [Table 1]. In GC1F, the presence of Asp (aspartic acid) rather than Glu (glutamic acid) at position 416 confers more negative charge (“acidity”) than GC1S, pulling GC1F more strongly toward the anode. In contrast to the GC1 variants, replacement of neutral Thr (threonine) with positively charged Lys (lysine) in GC2 results in the loss of an essential O-linked glycan (trisaccharide), which often ends with negatively charged sialic acid. As a result, GC2 migrates significantly more slowly toward the anode than both GC1 variants^[14]^ [Table 1].

### BIOLOGICAL BASIS AND PLEIOTROPIC FUNCTIONS OF GC

#### Molecular structure of GC: structure-activity relationships

GC likely arose from a gene triplication event^[15]^ and possesses three domains, one of which binds to vitamin D. Based on its crystal structure^[16–19]^ [Figure 1A], GC has an all alpha-helical structure with 10 helices in domain I (residues 1–191) and 9 helices in domain II (residues 192–378), while the third domain is truncated at the C-terminus and only contains 4 alpha helices (residues 379–458)^[19]^. The N-terminal part of domain I contains a cleft structure formed by helices 1–6 enabling high-affinity binding to vitamin D^[20]^ [Figure 1B]. Interestingly although rs7041 and rs4588 affect AAs in domain III, these single nucleotide polymorphisms (SNPs) are associated with vitamin D binding affinity^[21]^. Domain I also contains residues at AAs 130–149 that are essential for enhancing the chemotactic function of complement component 5-derived peptides (C5a/C5a^desArg^)^[22]^ [Figure 1C], although this activity does not involve direct binding to C5^[23]^. Residues spanning both domains I and III (AAs 150–172 and 379–402) enable binding to fatty acids in the cell membrane^[24]^ [Figure 1D]; mono- and polyunsaturated fatty acids interfere with vitamin D binding to GC, while saturated fatty acids do not^[25]^. This suggests competition for a shared binding pocket or allosteric modulation. Lastly, GC can bind to monomeric globular actin (G-actin)^[18,26]^ with high avidity (*K*_d_ = 2 × 10^9^ M^−1^) via a large binding surface of 3,600 Å^2^ that spans all three domains (helix 10 of domain I, helix 6 of domain II, and helix 3 of domain III)^[16,17,27–29]^ [Figure 1E]; actin binding does not directly interfere with vitamin D binding^[26,30]^. We summarize these structure-activity relationships in Table 2.

#### Classical functions and mechanisms of GC: the core transport protein in the vitamin D system

Vitamin D is a sterol needed for maintaining homeostatic serum levels of calcium in addition to bone development. GC is alternatively named for its role in binding vitamin D metabolites, and transports and sequesters the majority (85%–88%)^[31,32]^ of vitamin D in the body. According to the free hormone hypothesis^[33]^, lipophilic sterols such as vitamin D require their respective carrier proteins to travel through the circulatory system, but must be unbound and therefore free to enter cell membranes in order to act on their target tissues. In the liver, vitamin D from either sun exposure or dietary supplementation is converted into 25-hydroxy vitamin D [25(OH)D], which is the intermediate prohormone form in which the majority of vitamin D is transported within the organism. GC-bound 25(OH)D is taken up in the kidney via the megalin/cubilin transport system^[34,35]^, where it is further metabolized to its active form 1,25-dihydroxyvitamin D3 [1,25(OH)_2_D] in the proximal tubules^[34]^. GC binds to 25(OH)D with at least 10× higher affinity (*K*_d_ between 5 × 10^8^ M^−1^ and 1 × 10^11^ M^−1^)^[36–38]^ compared to 1,25(OH)_2_D (*K*_d_ = 4 × 10^7^ M^−1^)^[38]^. Once GC-bound 1,25(OH)_2_D reaches its target tissues, it dissociates from GC to enter cell membranes and govern transcription of vitamin D-responsive genes. Vitamin D binds to the nuclear transcription factor vitamin D receptor (VDR), which then forms a heterodimeric complex with retinoic acid X receptor (RXR). GC-bound vitamin D thus serves as a circulatory reservoir for local delivery of vitamin D^[39]^. Notably, only 1%–2% of sterol-binding sites on GC are occupied at any given time, suggesting GC’s primary biological role may not be limited to binding and transporting vitamin D^[36]^.

#### Non-classical functions and mechanisms of GC

GC plays pro-inflammatory roles based on its structure-activity relationships and post-translational modifications. First, GC behaves as an adjuvant, promoting inflammation by binding peptides derived from complement 5 (C5), one of the most potent activators of the inflammatory response^[40]^. Specifically, GC binds and enhances the chemoattractant functions of C5a and its stable degradation product C5a^desArg^ on phagocytic cells, including neutrophils^[41]^, monocytes/macrophages^[42]^, and fibroblasts^[42,43]^. GC itself does not have chemotactic functions^[23,40,44–46]^. Second, independent of its functional domains, GC can be converted into GCMAF (GC Macrophage Activating Factor)^[47,48]^ via O-glycosylation by B-cells and T-cells during inflammation^[49]^. Specifically, this occurs on threonine residues at positions 418 or 420 and GC1 carriers therefore have a higher capacity for GCMAF formation than GC2 carriers^[50]^. As its name suggests, GCMAF rapidly stimulates macrophage phagocytic activity^[51]^, and can also trigger macrophage death when these cells’ presence is no longer needed^[52]^. Interestingly, cancer cells can cause immunosuppression via loss of macrophage activation by releasing the enzyme alpha-N-acetylgalactosaminidase (alpha-NaGalase), which can deglycosylate GC and thereby remove the substrate needed for its conversion to GCMAF^[53]^. Third, GCMAF is involved in bone resorption, as its exogenous administration can rescue skeletal defects in rat models of osteoporosis caused by defects in the pathway involved in the conversion of GC to GCMAF^[54]^. Notably, this effect was found to be independent of vitamin D binding to GCMAF^[55]^, and subsequent studies implicated the glycosylation site in domain III of GC^[56]^.

During tissue injury-induced cell lysis, filamentous (F-) actin is released and severed by gelsolin into globular (G-) actin. According to the actin scavenger hypothesis^[57]^, GC binds and sequesters G-actin to prevent its spontaneous re-polymerization into F-actin that would otherwise damage and obstruct the microvasculature^[58]^, thereby exerting an anti-inflammatory effect. Depletion of GC would consequently be expected to damage small blood vessels and increase inflammation by reducing extracellular actin binding capacity. However, based on more recent experimental evidence, intravenous injection of G-actin results in less severe acute lung inflammation in Gc knockout (GcKO) mice compared to wild-type (WT) mice^[59]^. This result suggests that GC promotes inflammation in the presence of actin. Furthermore, consistent with this notion, the addition of GC-actin complexes to human endothelial cells causes inflammation and injury. These data indicate that the classical actin scavenger hypothesis is incomplete, and that GC promotes inflammation by forming GC-actin complexes. GC ablation is therefore expected to help reduce inflammation by preventing GC-actin complex formation.

### EVIDENCE LINKING GC TO METABOLIC DISEASES

#### GC genetic variation and metabolic diseases in humans

To corroborate the above *in vitro* and *in vivo* studies of GC, we next searched for human genetic associations between GC and human health and disease. Availability of human genetic evidence linking drug targets to diseases suggests causal relationships, and more than doubles the success rate of drug development^[60]^. Given the highly polymorphic nature of GC protein^[61]^, *GC* genotypes have long been investigated for associations with numerous human diseases, including cancer, chronic disease, infectious diseases, and neurodegenerative diseases, and the results of these studies have been systematically reviewed and summarized by others^[14,62–64]^. Many of these early studies generally featured small cross-sectional designs focused on specific variants such as rs7041 and rs4588 and were therefore less robust than larger and more comprehensive genome-wide scans for disease associations.

We therefore surveyed genome-wide association studies (GWAS) published and entered into the National Human Genome Research Institute - European Bioinformatics Institute (NHGRI-EBI) catalog as of August 25th, 2025, and found *GC*-associated phenotypes consistent with GC’s structure-activity relationships [Figure 2 and Supplementary Table 1]. Specifically, *GC* variants are associated with serum levels of GC (vitamin D binding protein)^[21,65–80]^ as well as vitamin D and its metabolites^[21,67,70,74,79,81–95]^, in addition to immune cell-related traits such as white blood cell counts^[65,79,96–101]^ and modulators of the immune response^[102,103]^. *GC* variants also have genome-wide significant associations with metabolic traits including blood lipid levels^[104–107]^, blood pressure^[108,109]^, bone mineral density^[110]^, and non-alcoholic fatty liver disease (NAFLD)^[111]^, neurological^[112–114]^ and other traits^[115–120]^, as well as serum levels of albumin^[121]^ and other proteins^[65,73,122–125]^.

We further queried GWAS data from population-scale biobanks consisting of electronic medical record phenotypes linked to genotypes [Supplementary Table 1]. In FinnGen Release 12, *GC* variants are significantly associated with phenotypes in the “Endocrine, nutritional and metabolic diseases” category including lipidemias, cholesterolemias, and metabolic disorders, in addition to statin medication prescription, height, cystic kidney disease, and diseases of the respiratory system^[126]^. In analyses of UK Biobank data by AstraZeneca, *GC* SNPs are significantly associated with vitamin D levels among Europeans and with vitamin D, reduced inflammation, whole-body impedance (fat mass), as well as reduced fat-free mass among Africans^[127]^. In summary, there is consistent human genetic evidence from large-scale studies linking *GC* with vitamin D metabolism, inflammation, and metabolic diseases.

#### Characterization of GC deficiency in humans and mice

Two homozygous *GC*-null individuals have been identified^[128,129]^. The first patient (Patient 1) was a 58-year-old Lebanese woman who carried two 139-kb deletions on chromosome 4q13.3 eliminating the entirety of *GC*, as well as nearby 144-kb deletions eliminating part of the neuropeptide FF receptor 2 (*NPFFR2*) gene^[129]^. The second patient (Patient 2) was a 60-year-old Pakistani man who had two copies of a c.702–1G>A variant, resulting in exon 7 skipping and the consequent loss of GC protein due to nonsense-mediated decay^[128]^.

Both patients had consanguineous parents (first and second cousins, respectively) and exhibited undetectable GC protein and very low 25(OH)D and 1,25(OH)D levels. In contrast, serum measures of albumin, calcium, phosphate, parathyroid hormone, alkaline phosphatase, as well as magnesium and calcitonin in Patient 1, were within normal clinical ranges. Neither patient exhibited bone metabolism or skeletal abnormalities associated with vitamin D deficiency, such as rickets, osteomalacia, or osteoporosis. Only Patient 1 had progressive ankylosing spondylitis as well as osteopenia and fragility fractures in the fifth decade of life, suggesting her low bone density and fractures were attributable to her rheumatic disease, rather than the apparent vitamin D deficiency^[128]^. The observed clinical differences between the two *GC*-null patients may therefore be attributable to genetic differences beyond the absence of *GC*, such as disruption of *NPFFR2* or nearby regulatory elements, and/or other genetic differences.

*Gc*-null mouse phenotypes generally mirror those of *GC*-null humans [Table 3], with plasma 25(OH)D and 1,25(OH)_2_D levels decreasing based on the dosage of inactive *Gc* and *GC* alleles while the remaining measurements available in both species are stable across genotypes. Mice with one (*Gc*^+/−^) or zero (*Gc*^−/−^) copies of functional Gc are viable, fertile, and do not exhibit any obvious phenotype compared to WT mice when placed on a normal (non-vitamin D deficient) mouse chow diet, despite a 95% reduction in serum 25(OH)D and 1,25(OH)_2_D^[38]^. Compared to WT mice, *Gc*-null mice only develop hyperparathyroidism and osteopathy (bone mineralization defects) secondary to vitamin D deficiency when placed on vitamin D-deficient diets. These mice also develop vitamin D deficiency symptoms more rapidly, likely due to accelerated 25(OH)D clearance in the absence of a reservoir of Gc-bound vitamin D^[38]^. Together, the evidence demonstrating that Gc-null mice phenocopy GC-null humans suggests that murine models of Gc deficiency are representative of GC deficiency in humans.

#### The function of Gc in mouse metabolic tissues

##### Gc in the pancreatic beta cell

With respect to metabolic diseases, *Gc* was initially identified as an effector gene in beta cell failure^[130]^. In healthy pancreatic beta cells, the expression of *Gc* is repressed at the transcript level by key beta cell transcription factors such as Foxo1. In contrast, the expression of Gc protein is increased during the progression of beta cell dedifferentiation and dysfunction. We next subjected mice to a metabolic challenge, in the form of a high-fat diet (HFD), to further investigate the role of Gc in beta cell function under obesogenic and diabetogenic conditions. Since we did not observe a significant deterioration of glucose tolerance in female mice, which are known to be more resistant to HFD challenges^[131–133]^, we focused our experiments on mice with diet-induced obesity. In hyperglycemic clamp experiments, which assess beta cells’ ability to secrete insulin by varying the rate of glucose infusion necessary to maintain (i.e., clamp) a high blood glucose concentration, male whole body GcKO mice displayed improved insulin secretory capacity compared to WT [Figure 3A]^[130]^. Furthermore, acute *Gc* knockdown in WT male mice on a HFD preserved circulating insulin levels^[134]^. Together, these data indicate that reducing *Gc* expression protects beta cell function and insulin secretion.

##### Gc in metabolic tissues

When challenged with a HFD, male GcKO mice showed a ~22% decrease in fasting glycemia compared to WT. These mice also demonstrated improvements in glucose tolerance based on intraperitoneal injection of a bolus of glucose, improvements in insulin sensitivity based on insulin tolerance tests, and reduced gluconeogenesis after a meal based on pyruvate tolerance tests [Figure 3A]. Moreover, compared to WT mice on HFD, GcKO mice displayed (i) ~15% reduced body weight; (ii) ~27% decreased fat mass; (iii) preserved muscle mass; (iv) ~17% reduced circulating non-esterified fatty acids; (v) ~35% reduced triglycerides; (vi) improved glucose uptake in skeletal muscle (~22%); (vii) improved glucose uptake in adipose (~30%); and (viii) suppression of hepatic glucose production by ~3 fold after refeeding [Figure 3A and B]. Notably, the weight loss in GcKO mice did not result from a reduction in food intake. Furthermore, acute ~70% knockdown of *Gc* in the liver was sufficient to recapitulate the phenotypes of chronic GcKO mice, as evidenced by improved glucose tolerance, preserved hepatic insulin signaling, and protected circulating insulin levels^[134]^.

We observed several phenomena that may underlie the insulin sensitizing effects of *Gc* inhibition. First, compared to WT, indirect calorimetry studies found that GcKO mice had increased energy expenditure, which may be partially explained by a modest but significant increase in mitochondrial brown fat uncoupling protein 1 (*Ucp1*) expression in white adipose tissue. Ucp1 is involved in generating heat in brown adipose tissue (non-shivering thermogenesis)^[135]^. Second, Gc may also protect against oxidative stress in the liver based on RNA-seq followed by Gene Ontology analysis^[134]^. Moreover, *Gc* ablation increases Ceramide kinase expression. Cerk upregulates Nuclear factor erythroid 2-related factor 2 (NRF2), which is essential to maintain redox homeostasis^[136]^. Third, several changes in hepatic gene expression with known metabolic effects provide further mechanistic explanation. GcKO liver showed reduced Cytochrome P450 family 8 subfamily B member 1 (*Cyp8b1*) expression, whose ablation leads to resistance to hepatic steatosis and insulin resistance^[137]^. GcKO liver also displayed lower levels of 11β-hydroxysteroid dehydrogenase type 1 (*Hsd11b1*), and its upregulation is associated with metabolic disorders and obesity in humans^[138]^. GcKO mice had decreased liver expression of Krüppel-like factor 15 (*Klf15*), which encodes a transcription factor that promotes gluconeogenesis^[139]^, and Cytochrome P450 2E1 (*Cyp2e1*), which encodes an enzyme that is elevated in NAFLD and nonalcoholic steatohepatitis (NASH)^[140]^. Lastly, we found improved insulin signaling in the liver, based on increased levels of Ak strain transforming (Akt)/Protein Kinase B (PKB) phosphorylated at serine 473, a mediator of insulin signaling.

As GC’s primary role has been recognized as a vitamin D transporter, does the absence of GC lower tissue-level vitamin D? *GC* deletion could eliminate the barrier preventing vitamin D from entering target tissues, as only the kidney can uptake vitamin D-bound GC via the megalin-cubulin complex, but at the same time, shorten the half-life of vitamin D by depleting its reservoir. Based on experiments conducted by Safadi *et al*., although GcKO mice have low levels of vitamin D metabolites in the bloodstream, they are normal in general^[38]^. Only when GcKO mice are intentionally placed on a vitamin D-deficient diet do they develop bone defects due to vitamin D deficiency. Therefore, *Gc* ablation most likely does not affect tissue-level vitamin D with sufficient vitamin D intake. Moreover, clinical trial evidence does not support a role for vitamin D in the development of diabetes, as randomized control trials of vitamin D supplementation in individuals at risk of developing diabetes found no significant association with diabetes incidence^[141–143]^.

The question of how GC directly promotes insulin sensitivity remains open. Beyond transporting vitamin D, GC could carry biochemical entities that confer insulin resistance; these entities would be eliminated by GC ablation. Alternatively, GC could promote insulin receptor signaling through an unknown intracellular mechanism, although only kidney cells possess the molecular machinery needed for GC uptake. Considering low-grade inflammation is associated with insulin resistance^[144]^, it is plausible that GC deficiency improves insulin sensitivity by blunting inflammatory cascades driven by GC-actin and GC-fatty acid complexes, to reduce hepatic oxidative stress and fatty liver disease. More work is necessary to test these hypotheses.

### EVALUATION AND PROSPECTS OF GC AS A POTENTIAL THERAPEUTIC TARGET FOR METABOLIC DISEASES

While GLP1RAs have dramatically improved management of metabolic diseases, treatment-associated side effects limit tolerability and dosing, indicating an unmet need for a drug that promotes insulin secretion and sensitivity as well as fat mass reduction, without reducing food intake or muscle mass. The effects of GLP-1 receptor (GLP1R) agonism and GC inhibition on type 2 diabetes and obesity have not been compared head-to-head, but the improved metabolic phenotypes observed in acute and incomplete knockdown of *Gc* in mice demonstrate the possible utility of targeting GC to treat metabolic diseases via a novel mechanism of action that avoids appetite suppression and preserves lean mass, and could potentially improve outcomes in GLP1RA non-responders.

To our knowledge, there are no publicly disclosed therapeutic agents that inhibit GC. GC is a circulating protein that has an *in vivo* half-life of 2.5–3 days ^[38]^ and is primarily expressed in the liver by hepatocytes^[134,145,146]^. Considering the evidence above demonstrating the insulin-sensitizing effects of GC inhibition in the liver, and to minimize any off-target effects given GC’s many functions, it would be prudent to use a liver-specific GC inhibitor to treat metabolic diseases. While *Gc*-null mice and *GC*-null humans were viable and fertile, and acute knockdown achieved metabolic improvements observed in knockout mice, potential long-term safety considerations for pharmacological inhibition of GC revolve around its roles in vitamin D homeostasis, bone resorption, and immunity. The design, dosing, and clinical testing of such agents and subsequent monitoring will need to be done with these safety concerns in mind and are areas of ongoing investigation.

#### Conclusions and future directions

Based on the available data, GC represents a promising novel therapeutic target to treat metabolic disorders such as obesity and diabetes. *Gc* inhibition in mice preserves beta cell function with improved insulin secretion^[130]^, and increases insulin sensitivity in the liver, skeletal muscle, and adipose^[134]^. Moreover, these mouse studies show that chronic *Gc* ablation leads to reduced body weight due to reduced fat mass, while maintaining muscle mass and bone mass^[38,134]^. Although GC has long been recognized for its role in vitamin D and metabolite transport, recent findings strongly suggest additional functions in metabolism. GcKO mice only showed bone defects associated with vitamin D deficiency or phenotypes associated with vitamin D toxicity when deliberately placed on diets with deficient or excessive vitamin D, respectively^[38]^. Similar observations were made in the case reports of *GC*-null humans^[128]^.

The metabolic health benefits of GC inhibition are now clear, and studies to illuminate the mechanisms underlying these improvements are underway. Future directions include identifying the effectors of Gc in metabolic tissues, and signaling pathways involved in mediating its role in metabolism. These findings will provide insights important for developing next-generation therapeutics for metabolic diseases.

## Supplementary Material

Supplementary Materials

## Figures and Tables

**Figure 1.**
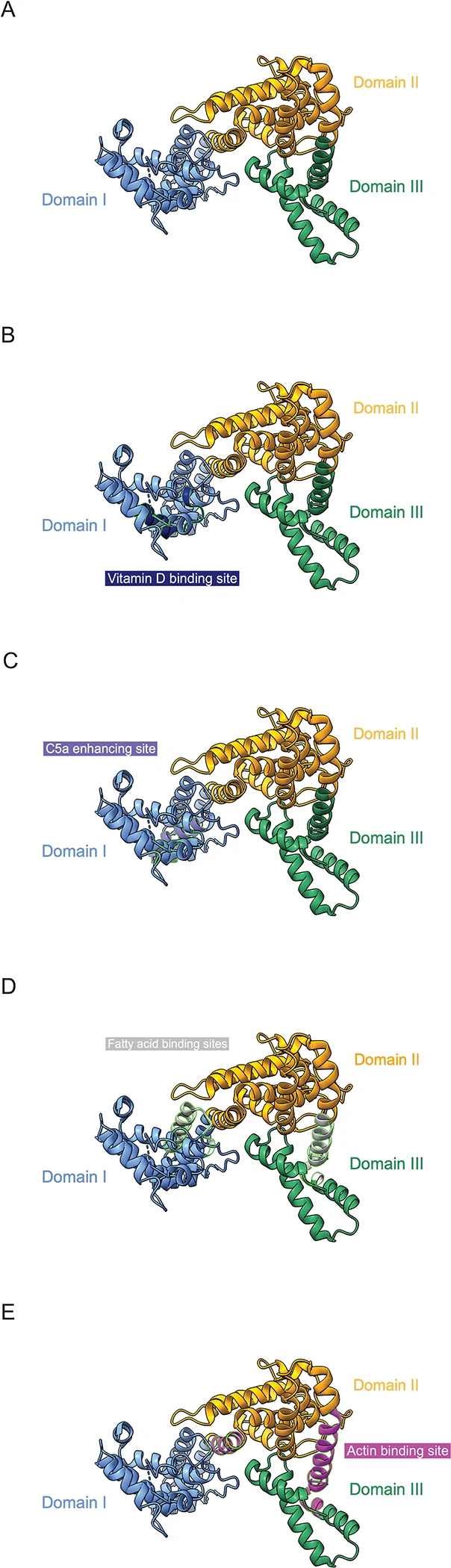
Three-dimensional structural diagram of GC protein and its domains (A). Subsequent panels highlight GC subdomains containing its vitamin D binding site (B), C5a enhancing site (C), fatty acid binding sites (D), and actin binding site (E). GC: Group-specific component; C5a: complement component 5a.

**Figure 2.**
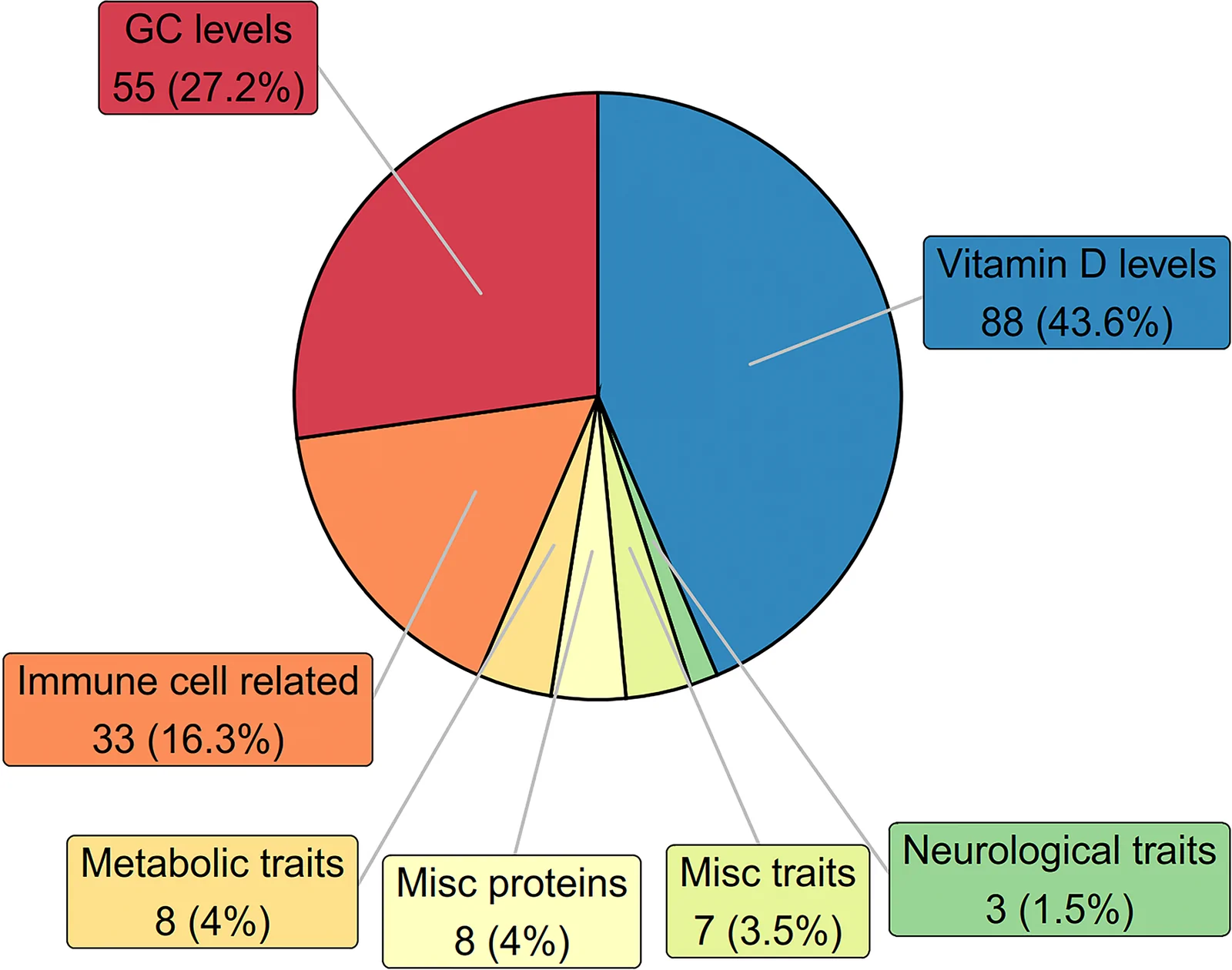
Number and proportion of *GC* variants associated with distinct groups of phenotypes in the NHGRI-EBI GWAS Catalog as of August 25th, 2025. https://www.ebi.ac.uk/gwas/genes/GC. GC: Group-specific component; NHGRI: National Human Genome Research Institute; EBI: European Bioinformatics Institute; GWAS: genome-wide association study.

**Figure 3.**
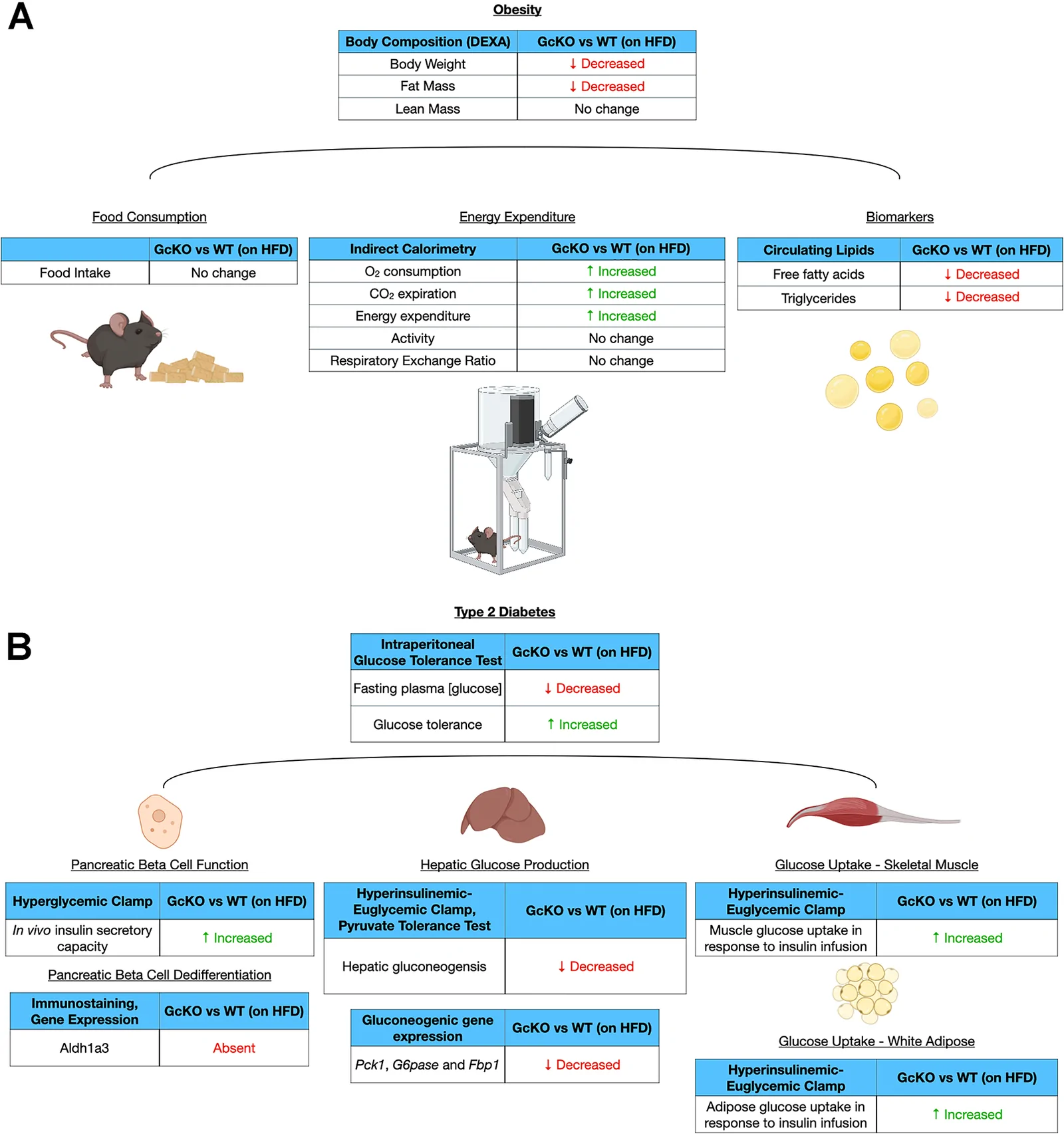
Effects of Gc inhibition on hallmarks of (A) type 2 diabetes and (B) obesity in high fat diet-fed mice, based on previous studies^[130,134]^. GcKO: Gc knockout mice. WT: Wild-type mice. HFD: High-fat diet. DEXA: dual-energy X-ray absorptiometry. Created in BioRender. Gill, R. (2026) https://BioRender.com/buhul0t. Gc: Group-specific component; GcKO: Gc knockout mice; WT: wild-type mice; HFD: high-fat diet; DEXA: dual-energy X-ray absorptiometry; Aldh1a3: aldehyde dehydrogenase 1 family member A3; *Pck1*: phosphoenolpyruvate carboxykinase 1; *G6pase*: glucose-6-phosphatase; *Fbp1*: fructose-1,6-bisphosphatase 1.

**Table 1.** The three major electrophoretic variants of GC protein

GC form	rs7041	AA 416	rs4588	AA 420

GC1F	T	Asp (D)	C	Thr (T)
GC1S	G	Glu (E)	C	Thr (T)
GC2	T	Asp (D)	A	Lys (K)

AA positions refer to residues in mature human GC protein. GC: Group-specific component; AA: Amino acid; GC1F: group-specific component 1F variant; GC1S: group-specific component 1S variant; GC2: group-specific component 2 variant.

**Table 2.** Human GC structure-activity relationships

Protein domain	AA	Helices	Function	Notes	Ref.

I	1–191	1–10			
II	192–378	1–6,8,9,10			[19]
III	379–458	1–4			
I	35–49	1–6	Vitamin D binding 25(OH)D: *K*_d_ = 5 × 10^8^ M^−1^−1 × 10^11^ M^−1^ 1,25(OH)_2_D: *K*_d_ = 4 × 10^7^ M^−1^	Cleft, in contrast with vitamin D receptor (closed pocket)	[20]
I	130–149	7,8	Enhance C5 chemotactic function	Not a C5 binding site	[22]
I	150–172	8,9	Fatty acid (cell membrane) binding		[24]
III	379–402	1,2			
I	194–207	10	G-actin binding: *K*_d_ = 2 × 10^9^ M^−1^	Does not interfere with vitamin D binding	[16,17,27–30]
II	373–403	6			
III		3			
III	418, 420		Threonine residues required for conversion to GCMAF via O-glycosylation	Primarily in GC1F/S, *vs*. GC2 carriers	[53]

AA positions refer to residues in mature human GC protein. GC: Group-specific component; AA: Amino acid; 25(OH)D: 25-hydroxyvitamin D; 1,25(OH)_2_D: 1,25-dihydroxyvitamin D; Kd: dissociation constant; C5: complement component 5; G-actin: globular actin; GC1F: group-specific component 1F variant; GC1S: group-specific component 1S variant; GC2: group-specific component 2 variant; GCMAF: GC macrophage-activating factor.

**Table 3.** Laboratory measurements in GC-null humans and Gc-null mice, compared to wild-type

	Clinical and biochemical data from studies of human *GC*-null cases					Phenotypic data from studies of *Gc*-null mouse models		

Study		Henderson *et al*. 2019^[129]^			Banerjee *et al*. 2021^[128]^	Safadi *et al*. 1999^[38]^		
Lab result	Normal clinical ranges	+/+ Sibling	+/− Sibling	GC-null Patient 1	GC-null Patient 2	WT mouse	+/− mouse	Gc-null mouse
Total plasma 25(OH)D ng/mL	20–100	19.655	13.139	0.079	1.07	34	21	3
Total plasma 1,25(OH)_2_D pg/mL	21.2–73.1	55.7	19.5	5.6	14.2	24	17	4
Free 25(OH)D pg/mL		3.2	3.4	1.8	3.45			
GC μg/mL	104–477	224.5	95.3	< 3.0	< 1			
Albumin (g/dL)	3.3–4.8			3.8–4.2	4.5			
Serum calcium (mg/dL)	8.4–10.2	9.9	9.66	9.58	8.9–10.2	8.0 ± 0.3		7.9 ± 0.8
Phosphate (mg/dL)	2.4–5.0	2.76	2.23	2.45	2.5–3.7	11.4 ± 1		10.3 ± 1.5
Parathyroid hormone (pg/mL)	12–90	43	42	57	54.1–94.2	566		633
Alkaline phosphatase (U/L)	37–117			30–115	52–68	74 ± 28		94 ± 26
Magnesium (mg/dL)	1.6–2.6			1.7–2.1				
Calcitonin (ng/L)	< 12			< 2				
Urinary Ca excretion (mg/d)	100.2–300.6			94–414	177.0 @55y 671.0 @59y			
Urinary Mg excretion (mg/d)	388.9–1,166.6			608				
Calculated urinary Mg (mg/d)	72–103				91			
Calculated urinary creatinine (mg/d)	1,000–2,000				2,000 @55y 2,024 @59y			

GC: Group-specific component; 25(OH)D: 25-hydroxyvitamin D; 1,25(OH)_2_D: 1,25-dihydroxyvitamin D; +/+: homozygous wild-type; +/−: heterozygous; WT: wild-type.

## Data Availability

Not applicable.
